# Brain Neoplasms and Coagulation—Lessons from Heterogeneity

**DOI:** 10.5041/RMMJ.10164

**Published:** 2014-10-29

**Authors:** Esterina D’Asti, Yi Fang, Janusz Rak

**Affiliations:** Department of Pediatrics, McGill University. Montreal Children’s Hospital, The Research Institute of the McGill University Health Centre, Montreal, QC, Canada

**Keywords:** Brain cancer, coagulation, dormancy, glioma, medulloblastoma, oncogenes

## Abstract

The coagulation system constitutes an important facet of the unique vascular microenvironment in which primary and metastatic brain tumors evolve and progress. While brain tumor cells express tissue factor (TF) and other effectors of the coagulation system (coagulome), their propensity to induce local and peripheral thrombosis is highly diverse, most dramatic in the case of glioblastoma multiforme (GBM), and less obvious in pediatric tumors. While the immediate medical needs often frame the discussion on current clinical challenges, the coagulation pathway may contribute to brain tumor progression through subtle, context-dependent, and non-coagulant effects, such as induction of inflammation, angiogenesis, or by responding to iatrogenic insults (e.g. surgery). In this regard, the emerging molecular diversity of brain tumor suptypes (e.g. in glioma and medulloblastoma) highlights the link between oncogenic pathways and the tumor repertoire of coagulation system regulators (coagulome). This relationship may influence the mechanisms of spontaneous and therapeutically provoked tumor cell interactions with the coagulation system as a whole. Indeed, oncogenes (EGFR, MET) and tumor suppressors (PTEN, TP53) may alter the expression, activity, and vesicular release of tissue factor (TF), and cause other changes. Conversely, the coagulant microenvironment may also influence the molecular evolution of brain tumor cells through selective and instructive cues. We suggest that effective targeting of the coagulation system in brain tumors should be explored through molecular stratification, stage-specific analysis, and more personalized approaches including thromboprophylaxis and adjuvant treatment aimed at improvement of patient survival.

## INTRODUCTION

The vascular system plays a unique and consequential role in brain homeostasis, function, and pathology. Due to perfusion demands, high-energy metabolism, and endocrine signal exchange, blood supply to the brain parenchyma (neuropil) is programmed to be high, uninterrupted, and tightly controlled.^1^ While the brain represents only 2% of the body mass, it receives 20% of cardiac output, resulting in the flow rate of approximately 50 mL of blood per 100 g of tissue every minute.^2^ The vasculature of the central nervous system (CNS) evolved to meet these unique demands in multiple ways, including through highly regulated perfusion, transendothelial transport mechanisms, and protective effects of the blood–brain barrier (BBB). In addition, the absence of the lymphatic circulation in the brain places demands on the vascular system in terms of the interstitial fluid circulation pattern^3^ and mitigation of physical stress on tissues locked within the rigid confines of the intracranial space. Finally, the regulation of vascular integrity, patency, and sustained blood supply depends on specialized mechanisms of hemostasis regulation adapted to the brain microenvironment.^4^

Intracranial dysfunction of hemostatic processes triggers profound local and systemic consequences, often with lethal outcomes including thrombosis, local vascular occlusion, hypoxic tissue damage, coagulation factor neurotoxicity, ischemic stroke, hemorrhage, and edema.^4^ Notably, thrombotic events occurring locally in the brain often coincide with peripheral coagulopathy through mechanisms that are not fully understood.^4^ Nonetheless, a distinct vascular microanatomy in the brain creates several regulatory points that may make contact with the hemostatic system in blood and may become involved in disease. For example, neurons receive trophic inputs from blood through astrocytic glial cells, which make contact with capillaries through their specialized foot processes. Interestingly, studies with mice indicate that these structures may be the body’s richest sources of tissue factor (TF), which acts as the key initiator of the coagulation cascade.^4^,^5^ The physiological role of TF in the brain is presently uncertain and possibly minimal as documented by the absence of overt anomalies in mice with brain-specific disruption of the TF/F3 gene (Pawlinski, unpublished observations). However, the ability of astrocytes to express TF may have profound consequences in vascular pathology, including in cancer.

## INTERACTIONS BETWEEN THE COAGULATION SYSTEM AND BRAIN PARENCHYMA

Pathological disruption of the vascular continuity and barrier function in the brain leads to formation of abnormal contact points between coagulation proteases, cell surfaces, and extracellular matrix present in the neuropil. In this regard, TF acts as a high-affinity receptor/co-activator for the blood-borne coagulation factor VII/VIIa and thereby functions as the main trigger of the coagulation cascade. Formation of the TF/VIIa complex activates factor X (to Xa) and leads to the generation of thrombin (IIa), which catalyzes the formation of fibrin and activation of platelets, as well as triggers the amplification phase of the coagulation cascade. These mechanisms lead to the deposition of fibrin matrix, clots, platelets, growth factors, and enzymatic activities within the intravascular and perivascular microenvironment.^6^ While these processes are programmed to lead to a rapid and self-contained hemostatic response followed by the clot resolution, they could become protracted in chronic disease states such as tumor formation. This, in turn, could result in vascular occlusion, ischemia, thrombin-mediated neurotoxicity, and cellular (non-coagulant) effects exerted by mediators of coagulation and fibrinolysis.^4^,^7^

The coagulation system evolved for over 450 million years^8^ to become the most immediate form of tissue responses to damage. Processes of clot formation and resolution are closely integrated with inflammation, angiogenesis, stromal cell recruitment, and tissue repair,^9^–^12^ so much so that coagulation system effectors may directly participate in the modulation of inflammatory and angiogenic responses.^7^,^13^–^16^ Various vascular and brain cell populations are equipped to respond to hemostatic and fibrinolytic proteins through molecular sensors such as TF and thrombin receptors (PAR-1), other protease-activated receptors (PARs), urokinase receptor (uPAR), thrombomodulin (TM), endothelial protein C receptor (EPCR), and receptors for protein S (TAM family kinases, e.g. AXL). In addition, integrins and growth factor receptors may cooperate with coagulation pathway signaling through trans-activation, or participation in their related cellular effects and changes in gene expression.^6^,^17^–^20^ These interactions explain the unexpected complexity of defects observed in mice with disruption of coagulation system effectors^21^ and should be considered as factors in brain pathologies including the role of the clotting pathway in the formation and progression of primary and metastatic brain cancers.^22^,^23^

## THE INVOLVEMENT OF THE COAGULATION SYSTEM IN CANCER

The various facets of the coagulation system are persistently challenged during the development of human cancers.^24^ This is manifested as a spectrum of well-recognized co-morbidities including hypercoagulability, venous thromboembolism (VTE), pulmonary embolism (PE), localized vaso-occlusion, coagulation factor consumption, disseminated intravascular coagulation (DIC), hemorrhage, and several other states of considerable medical concern.^24^,^25^ Unexplained coagulation disorders may be indicative of an occult malignancy (Trousseau syndrome),^26^ or arise in cancers already diagnosed, leading to significant medical needs.^25^ Indeed, thrombosis is the second leading cause of cancer-related deaths^27^ and poor outcomes,^28^,^29^ aspects that could be linked to both hematological and noncoagulant (biological) effects of the coagulation system, possibly including processes such as angiogenesis, inflammation, growth, invasion, and other changes in cellular phenotypes.^17^,^30^–^32^ The emerging early evidence suggests that in certain forms of congenital thrombophilia, such as homozygous factor V Leiden mutations, the incidence of colorectal cancer can increase as much as 6-fold,^33^ while genetic targeting of coagulation factors in mice may impact inflammation-driven experimental tumorigenesis in the gut.^34^ Conversely, cancer progression almost always leads to hemostatic perturbations, which accompany nearly 90% of metastatic malignancies for reasons that may seem intuitively obvious, but are often mechanistically elusive.^25^,^35^,^36^

## FACTORS CONTRIBUTING TO CANCER COAGULOPATHY

How could coagulation system perturbations be triggered in cancer? Although the related events are likely context-specific, there are at least three major components of possible relevance to cancer coagulopathy that are worthy of more thorough consideration. First, vascular homeostasis is chronically challenged by the disruption of the tissue architecture associated with tumor growth, including vascular invasion and compression, persistent angiogenesis, chronic inflammation, extravasation of bone marrow-derived cells (BMDC), and metastatic entry of cancer cells into the vascular space. These events expose potentially procoagulant cells to coagulation factors in plasma. In addition, the breakdown of vascular barriers enables the uninhibited release of non-cellular material (metabolites, soluble factors, cytokines, cellular debris, and extracellular vesicles) from the tumor mass into the blood stream. Thus the chronically compromised integrity of the vessel wall, intermittent hemorrhage, increased vascular permeability, extravasation of plasma proteins, and activation of clotting factors through contact with coagulant surfaces of extravascular cells could collectively act as a “structural” trigger of the coagulation system in agreement with the known tenets of the Virchow triad (endothelial damage, stasis, and hypercoagulability).^24^,^37^

Second, coagulation system perturbations may be triggered by the effects of anticancer therapy. For example, therapeutic interventions such as surgery, radiation, systemic administration of chemotherapeutic and antiangiogenic agents, placement of central venous lines, and protracted stasis due to bed rest may create procoagulant conditions.^25^ Exaggerated or unopposed iatrogenic coagulopathy could become a source of considerable morbidity,^38^ mortality,^27^ and adverse outcomes.^28^,^29^ Indeed, the pressing question remains whether the immediate benefit of therapeutic interventions may be at times offset, at least to some extent, by belated coagulation-dependent processes that could influence long-term progression and disease outcomes.

Third, a part of cancer biology may entail a procoagulant conversion of the tumor and stromal cell phenotype. Deregulation of cellular signaling pathways due to microenvironmental perturbations, hypoxia, exposure to inflammatory cytokines, and other factors may drive expression of coagulation-related genes and exaggerated procoagulant, anticoagulant, or fibrinolytic properties of tumor cells and their associated stroma. High cellular turnover rate, necrosis, and increased activation of cell death pathways may lead to the exposure of phospholipids and TF on the surface of dying cancer, inflammatory, endothelial, and stromal cells, leading to their increased procoagulant activity.^39^ Release of cellular DNA by vesiculation and NET-osis (formation of neutrophil extracellular traps composed of DNA), either spontaneously or due to exposure to cytotoxic therapy, may also contribute to these events in ways that are only beginning to be understood.^40^–^42^ Importantly, the very malignant progression of cancer cells themselves may impact their expression of coagulation-related genes, linking changes in the intracellular genome to the state of the (cellular, pericellular, and systemic) “coagulome.”^24^,^25^,^35^,^36^,^43^–^47^

## CANCER-SPECIFIC FACTORS THAT MAY IMPACT COAGULOPATHY

Procoagulant complications in cancer patients have long been considered to be “unspecific” side effects of the underlying malignancy, or undesirable aftermaths of the related care. Consequently, the development of thromboprophylaxis and treatment approaches has largely been guided by considerations predicated on the state of the hemostatic equilibrium, and the assessment of clinical symptoms and risks in various disease contexts.^25^,^48^ In this sense, the use of low-molecular-weight heparin (LMWH) or oral anticoagulants was not diversified on the basis of the tumor type or biology in a given patient, but instead was based on general medical considerations.^48^

However, the intrinsic risk of thrombosis varies greatly between different cancers, including their site of origin and stage of progression.^49^ For example, such risk is remarkably elevated in high-grade astrocytic brain tumors, especially glioblastoma multiforme (GBM), exocrine pancreatic ductal adenocarcinoma (PDAC), and ovarian cancer, but far less severe in breast, prostate, or skin cancers.^49^–^51^ Moreover, the nature of the risk may differ, as in PDAC and GBM the coagulopathy is manifested mainly as venous thromboembolism (VTE) including pulmonary embolism (PE); while in acute promyelocytic leukemia (APL) the predominant alteration involves bleeding due to consumptive disseminated intravascular coagulation (DIC).^24^,^52^ Interestingly, the thrombotic risk in the pediatric cancer patient population is considerably different than in corresponding adult malignancies.^53^ This notion is exemplified by a paradoxically low event rate in pediatric GBM (pGBM) as compared to adult cases (aGBM), in spite of similar tumor histology, location, expression of clotting factors, florid angiogenesis, and the presence of intratumoral thrombi.^53^–^55^ It is also of interest that, in APL, restoration of cancer cell differentiation capacity through the therapeutic use of all-trans retinoic acid (ATRA) also modulates thrombosis and results in a marked downregulation of TF by leukemic cells.^52^ In addition, in pancreatic, colon, intestinal, brain, and other malignancies the expression of TF and the related coagulant potential increase with tumor grade,^35^,^56^,^57^ an observation suggesting that the intrinsic programs of malignant transformation may contribute to the expression of this aspect of the procoagulant phenotype, and perhaps others as well.^43^

## ONCOGENIC PATHWAYS AND THE COAGULANT PHENOTYPE OF CANCER CELLS

We have initially proposed that oncogenic transformation may have a role in triggering cancer coagulopathy.^58^ Indeed, molecular aberrations (mutations) driving human cancers possess unique molecular features and phenotypic consequences,^59^ and so could their impact on the ability of tumor cells to interact with the coagulation system.^43^ Driver mutations alter or abolish the function of specific genes acting as tumor suppressors (e.g. PTEN, TP53, SMAD4) or result in activation of proto-oncogenes (RAS, MYC, MET, EGFR). This may lead to un-scheduled or exaggerated activation of the respective signaling functions. However, oncogenic mutations may also act more broadly by affecting the function of multiple genes through their impact on the cellular epigenome (ATRX, H3FA3), genetic stability and DNA repair (MLH1, MSH2, TP53), cellular replication potential (TERT), protein translation (EIF4E), or stemness/ differentiation (NOTCH, WNT).^59^ In all these instances, the changes in expression of the respective target genes may include regulators of the vascular system, such as angiogenic factors (VEGF), angiogenic inhibitors (TSP1), and inflammatory mediators (IL8),^60^,^61^ all of which could trigger processes capable of modulating coagulation indirectly.^43^ For example, deregulation of angiogenic mediators results in formation of aberrant vascular networks^62^ as well as hyper-permeable, procoagulant,^63^ and incomplete endothelial lining.^37^ The mechanisms and nature of these effects differ between specific cancers, at least in part due to their genetic profiles, and also in relation to vascular properties of the affected organs.^60^,^64^

Oncogenic mutations may also influence coagulation more directly.^43^,^65^ Thus certain coagulation-related genes, such as TF, are regulatory targets of oncogenic signaling pathways, and their expression could be abnormally elevated in cancer cells.^15^ Moreover, transforming signals may trigger the ectopic expression of coagulation-related genes in cancer cells,^47^,^66^,^67^ or stimulate production of cytokines and extracellular vesicles (EVs) capable of modulating coagulant phenotypes of adjacent or distant tumor cell and stromal cell populations.^68^ These events have been documented in the case of driver mutations affecting RAS genes and in elements of the RAS signaling pathway.^36^ Similar findings have been recorded in relation to epidermal growth factor receptor (EGFR),^67^,^69^ HER2/ErbB2 proto-oncogene,^70^ MET receptor,^45^ as well as several tumor suppressors such as TP53 and PTEN,^36^,^44^ as recently reviewed elsewhere.^71^ Indeed, while the organ site, therapy, and other factors may play fundamentally important roles in triggering cancer coagulopathy,^25^,^72^ the net result may also be influenced by the emerging link between oncogenic events in cancer cells at their primary or metastatic sites, and by the related changes in the tumor coagulome.^65^

## GENETIC EVOLUTION AND HETEROGENEITY OF TUMOR CELL POPULATIONS—IMPLICATIONS FOR CANCER COAGULOPATHY

The notion that cancer-specific transforming mutations may impact the coagulome of tumor cells and their ability to reprogram vascular micro-environment is consistent with the observed variation amongst human cancers in terms of the risk of the associated coagulopathy.^49^ One aspect of this interrelationship that remains poorly studied, are the implications of the inter- and intratumoral heterogeneity of cancer cells (clonal, spatial, and temporal). Do cancer cell subsets differ with respect to their coagulant phenotypes and what are the determinants?

**Figure 1.**
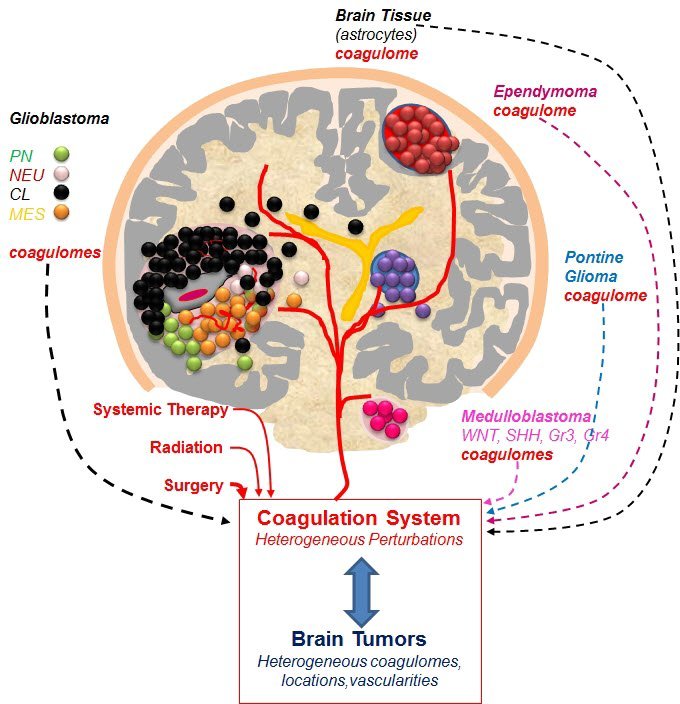
**Heterogeneity of Brain Tumors as a Possible Source of the Heterogeneous Nature of the Related Coagulopathies.** Brain is normally shielded from the coagulation system effectors by the blood–brain barrier (BBB), especially through the properties of the vascular wall. This barrier can be disrupted by injury or chronic pathology, such as cancer, and anti-cancer therapies (e.g. surgery), resulting in contact between brain parenchyma and coagulation factors in blood plasma. We postulate that these interactions could be more heterogeneous than currently thought and may lead to different mechanisms and consequences of coagulation system activation in specific pathological settings. Thus, cancers originate in different regions of the brain, where cells may possess different (currently unstudied) abilities to activate or respond to coagulation factors. Moreover, different brain tumors emerging in such distinct locations may differ in their abilities to activate coagulation. For example, such effects are pronounced and systemic in glioblastoma (GBM), but more subtle and mostly clinically unremarkable in medulloblastoma (MB). In addition, within specific tumor types, such as GBM, recent studies distinguished several molecular subtypes, such as proneural (PN), neural (NEU), classical (CL), and mesenchymal (MES) disease (symbols as indicated). Recent studies revealed that each of these subtypes expresses different repertoires of coagulation effector genes (coagulome).^47^ Similar diversity in coagulome has also been documented among subtypes of MB, such as wingless (WNT), sonic hedgehog (SHH), group 3 (G3) and group 4 (G4) tumors each driven by different oncogenic pathways. In MB more subtle interactions with the coagulation system may lead to subclinical effects. Finally, each tumor (especially GBM) may contain microregions and diverse cellular subpopulations that may have different coagulant properties. We propose that these factors may need to be considered when exploring the role of the coagulation system in brain tumor biology and the associated coagulopathy (see text).

Thus the vast majority of adult human cancers arise as a result of the accumulation of multiple genetic hits. As the “founder mutation” is compounded by additional mutational events, multiple cellular lineages with different genetic profiles emerge within a single lesion often colonizing different tumor microregions, or coexisting in dynamic mixtures composed of cells with different degrees of aggressiveness.^59^ Tumors emerging within the same organ site may possess similar histology but differ markedly from each other in terms of their mutational repertoires, such that they could be classified into distinct molecular subtypes of what once may have been thought to be a single diagnostic entity (e.g. GBM). Multiple human tumors exhibit such molecular heterogeneities, including breast, colon, and brain tumors.^73^–^75^ Notably, stromal and vascular properties of certain cancers may also serve to distinguish disease subtypes and pathological trajectories,^76^ a property that could impact coagulopathy, but has not been studied or discussed in this context (Figure 1).

Even within the same lesion, cancer cells that possess the same repertoire of genetic alterations are not phenotypically identical. Indeed, the disease-causing potential is thought to reside in a subset of cells harboring certain stem cell markers and referred to as tumor-initiating (stem) cells (TICs). These cells differ from their more differentiated but genetically related progeny with respect to gene expression profile, self-renewal, proliferative activity, and resistance to genotoxic insults.^77^ Recent studies pointed to variation between TIC and ‘bulk’ cancer cells in terms of their ability to interact with the coagulation system,^78^ including differential TF expression,^79^ growth in fibrin gels,^80^ and positivity for endothelial protein C receptor (EPCR).^46^ While in some tumors TICs appear to represent a minority of cancer cells,^81^ in other settings they are more prevalent in numbers, a property that may also influence their impact on the vascular and coagulation states in the respective cancers.^82^

As different TICs continue to acquire additional mutations their harboring cancers evolve to contain increasingly complex phylogenetic hierarchies of heterogeneous cellular clones.^83^ The composition of these populations is a function of their intrinsic diversity and the rate of change, enforced by cycles of microenvironmental or therapeutic selection.^84^ Again, recent studies suggest that cancer cell clones harboring different mutational and epigenetic repertoires (e.g. DNA methylation patterns) may occupy different microdomains in the same tumor, or dominate individual metastatic deposits.^85^

In this context, it could be argued that cancer coagulopathy may not only differ between tumor types^49^ but also between subsets of the same disease or between microdomains within a given tumor, resulting in a more complex pattern than hitherto realized. To reiterate, if oncogenic mutations influence the coagulome of specific cancers (directly or indirectly) the emerging heterogeneity would lead to at least two predictions. First, it would be expected that different molecular tumor subtypes (driven by different genetic events) may differ in terms of mechanisms (if not magnitudes) of their interactions with the coagulation system. Second, it is possible that within a single tumor the subclones of cancer cells and distinct tumor regions may exhibit different coagulant phenotypes (Figure 1). Some of these predictions are, indeed, borne out in the analysis of brain tumors, while others await further verification.

## HETEROGENEITY OF ONCOGENIC PATHWAYS IN HUMAN BRAIN TUMORS

There are significant unmet needs regarding clinical management of both primary (PBT) and secondary (metastatic) brain tumors (SBTs).^86^,^87^ The incidence of PBTs in the population is in the order of 2.8–3.7 per 100,000 and is markedly higher in the developed world.^86^ These biologically, histologically, and clinically diverse tumors can be classified into several major groups on the basis of their histology, location, and clinical features, including astrocytoma, ependymoma, meningioma, and embryonal tumors (e.g. medulloblastoma or embryonal tumor with multilayered rosettes) (Figure 1).^88^–^90^ In addition, the grading system has been developed to distinguish histologically and clinically indolent tumors from their more aggressive, high-grade counterparts. Of those the most common in adults are astrocytic glial tumors (gliomas), which have been divided into either low-grade diseases (LGG, grades I and II), or high-grade infiltrative gliomas (HGG) including anaplastic astrocytoma (AA) and glioblastoma multiforme (GBM). Glioblastoma multiforme exhibits a number of unique morphological characteristics such as cellular atypia, proliferative and invasive behavior of cancer cells, prominent hypoxic regions coupled with pseudopalisading necrosis, as well as exuberant angiogenesis, vascular proliferation, and intravascular thrombosis.^91^

While the histological features of GBM are relatively consistent between different cases, genetic studies revealed the existence of several molecular pathways driving this disease as a function of age and repertoire of oncogenic mutations.^92^ For example, in younger patients, GBM may be characterized by mutation of the isocitrate dehydrogenase 1 gene (IDH1), which could be coupled with mutation of the TP53 tumor suppressor in the pathway leading to LGG, AA, and secondary GBM. Alternatively, IDH1 mutant cells could sustain chromosomal loss of the 1p19q region and give rise to oligodendroglioma.^92^ However, the more common pathway (95% cases) leading to GBM is activated in older patients without the preceding LGG (primary GBM) and results in mutations of telomerase promoter (*TERT*), amplification of chromosome 7 (*EGFR* gene), activating mutation of EGFR (*EGFRvIII*), and loss of chromosome 10 region (*PTEN*) amidst other changes.^92^ In contrast, children with histologically similar GBM exhibit mainly changes in genes that control the cellular epigenome and chromatin architecture (*H3FA3*, *ATRX*).^93^

In addition to this genetic diversity, the gene expression and methylation profiling of GBM through the efforts of the Cancer Genome Atlas (TCGA) Consortium resulted in the subdivision of these tumors into at least four molecular subtypes: proneural (PN), neural (NEU), classical (CL), and mesenchymal (MES) (Figure 1).^75^,^93^,^94^ Of those, the PN-type GBMs are characterized by expression of stem cell markers (CD133, SOX2), MES GBMs exhibit pronounced inflammatory and stromal features, while CL tumors upregulate EGFR.^75^ Although individual cells isolated from GBM may exhibit some features of different subtypes,^95^ the molecular classification has set a new paradigm for the diagnosis of these lethal tumors and provided an informative framework for properly stratified therapeutic studies in the clinic.

Similar efforts are ongoing in several other types of brain malignancies of which perhaps the most advanced is the molecular classification of primitive neuroectodermal tumors including medulloblastoma (MB), tumors occurring mainly in the cerebellum.^96^ These primarily pediatric tumors are now known to consist of at least four different molecular subtypes described as WNT, SHH, Group 3, and Group 4, a classification that brought about significant translational and therapeutic consequences.^97^ For example, the molecular signature of the Wingless signaling pathway in WNT tumors correlates with favorable prognosis, susceptibility to surgical treatment, and benefits from de-escalation of debilitating radiation therapy previously administered to all MB patients. In contrast, the signature of sonic hedgehog signaling in SHH tumors signifies intermediate prognosis and high likelihood of MET receptor activation, while Group 3 tumors are the most lethal, especially when harboring amplification of the MYC proto-oncogene.^96^ Other mutations have also been described in other primitive neuroectodermal (PNET)-like tumors, including the amplification of the oncogenic microRNA cluster on chromosome 19 (CM19C) in a rare but aggressive form of brain malignancy known as embryonal tumor with multilayered rosettes (ETMR).^98^ This comparison illustrates the astounding molecular diversity of malignancies occurring within a similar organ site and often clustered together in clinical studies (Figure 1).

These and other advances in the molecular pathology of brain tumors have rarely been considered in the context of the coagulome. While exuberant vascular features, hypoxia, and upregulation of VEGF in GBM have attracted considerable interest and led to experimental and clinical explorations of antiangiogenic therapy,^99^ the underlying context of oncogenic pathways have not been fully explored, and this is true also for other primary and metastatic brain tumor types.^100^ Moreover, in spite of the reportedly high rate of thrombosis in GBM patients^29^ and the emerging preclinical results linking oncogenic pathways to the coagulome,^47^,^101^ these questions have not been widely considered in the clinical literature, or led to studies involving molecularly stratified patient cohorts.

## THE LINK BETWEEN ONCOGENIC HITS AND CHANGES IN BRAIN TUMOR COAGULOME

Two classes of factors may influence the procoagulant potential associated with brain tumors, the nature of the brain milieu, and the intrinsic molecular characteristics of tumor cells themselves. Thus the brain microenvironment presents an a-priori heightened procoagulant activity due to the concentration of TF on the surface of astrocytes, but also due to other factors that still need to be identified.^4^ This may result in the exacerbated systemic risks of thrombosis in association with brain surgery (3%–20%),^29^ injury, or disease, regardless of its intrinsic nature.^4^ For example, CNS lymphoma is associated with higher thrombotic potential than extra-cranial presentation of a similar malignancy (Benjamin Brenner, personal communication).^72^ It is of interest whether different regions of the brain (e.g. supratentorial or infratentorial sites) possess the same or different abilities to interact with the coagulation system in disease, and whether tumor location in them predicts the systemic risk of thrombosis.

There is, however, mounting evidence that the profiles of coagulation effectors change with genetic progression of human brain tumors. It is important to note that while this may impact the intrinsic risk of clinically detectable thrombosis, changes in the cancer cell coagulome may also have other more subtle and context-dependent consequences. For example, the expression of either a procoagulant or fibrinolytic cellular phenotype may, at least in theory, alter the responses to surgical excision of the respective lesions, leading to changes in iatrogenic clotting or bleeding tendencies, respectively. Moreover, even in the absence of clinically detectable hemostatic perturbations, unopposed activation of the coagulation system due to intervention or disease progression may impact tumor cell growth/quiescence equilibrium,^101^ dissemination,^102^ pro-inflammatory properties, and angiogenesis.^101^ In this regard, the data are relatively scarce especially with regard to metastatic brain tumors, but also in primary brain malignancies, especially as it relates to molecular underpinnings of coagulation. However, the aforementioned progress in molecular classification of GBM^75^,^92^ and MB^96^,^97^ could serve as a paradigm to illustrate the molecular links between oncogenic pathways and the cancer cell coagulome.

Progression of astrocytomas is linked to profound changes in coagulant properties of the respective tumors. For example, the risk of systemic thrombosis is high and continuous in GBM patients (1.7%–2.0% VTE per month of survival, or 17% at 6 months^29^). This is less pronounced in low-grade tumors and even less in pediatric GBM.^53^ Moreover, GBM, but not other brain tumors, exhibits a very high rate of thrombotic vaso-occlusion within the tumor bed (above 90%),^57^ and this correlates with areas of hypoxia and necrosis characteristic for this malignancy regardless of age.^103^ Whether intravascular thrombosis is a cause or a consequence of necrotic changes is presently unknown, and the link between these events and peripheral VTE is unclear.^104^ Although tumor microthrombi in anaplastic astrocytoma (which progresses to GBM) do not predict VTE, they are associated with poor survival, suggesting a link between coagulation and disease aggressiveness.^104^

While the molecular underpinnings of interactions between glioma cells and coagulation system are still poorly understood, brain tumor cells activate and respond to stimulation with clotting factors (VIIa, IIa) and PAR activating peptides.^23^,^67^,^102^ This property may depend on the availability of the respective receptors such as TF on the cancer cell surface. For example, GBM lesions reportedly express higher levels of TF mRNA than lower-grade astrocytomas.^104^–^107^ This may in some cases be paralleled by the upregulation of TF antigen *in situ*, and by the release of TF-containing extracellular vesicles into the circulation^108^ (unpublished observations); however, the consistency and magnitude of these events still remain controversial.^109^,^110^

Nonetheless, the causal relationship between oncogenic transformation and changes in the coagulome of glioma cells is well documented in preclinical studies. Thus the expression of the GBM-specific mutant of EGFR (EGFRvIII) in the U373 glioma cell line and in astrocytic cultured cells results in a dramatic upregulation of TF mRNA, protein, procoagulant activity, and proangiogenic signaling.^67^,^69^,^111^ Another common genetic hit in GBM resulting in the loss of PTEN expression is also associated with TF upregulation, which is exaggerated under hypoxic conditions.^44^ It is unclear whether other drivers of gliomagenesis (TERT, IDH1, 1p19q deletion, or H3FA3 mutations) impact TF or the coagulome directly or indirectly, but among those changes loss of TP53 appears to co-operate with the RAS pathway in driving TF expression in epithelial cells,^36^ and MET upregulates TF in medulloblastoma.^112^ Moreover, oncogenic EGFRvIII triggers the expression of other elements of the TF coagulation pathway such as FVII, PAR-1, and PAR-2, and potentiates the effects of TF/PAR signaling.^67^

## MOLECULAR SUBTYPES OF BRAIN TUMORS AS DETERMINANTS OF COAGULOME

The relationship between oncogenic drivers and the coagulome are not restricted to cultured cells. Notably, interrogation of transcriptome data sets compiled through the TCGA-sponsored analysis of GBM tumor samples reveals that the aforementioned molecular subtypes of GBM exhibit vastly different profiles of coagulation-related gene expression.^47^ This is in spite of histological similarity between these tumors, including vascular hallmarks of GBM such as proliferative endothelial cells and intravascular thrombi.^103^ In particular, elevated EGFR expression in the CL subtype of GBM closely correlates with upregulation of TF and PAR-1 transcripts in this tumor subtype, a feature not observed in MES, PN, or NEU-type GBM.^47^ The analysis of over 30 coagulation-related genes suggests that the MES subtype of GBM is relatively rich in fibrinolytic system effectors and endogenous anticoagulants, such as thrombomodulin (TM), tissue factor pathway inhibitors 1 and 2 (TFPI1/2), activated protein C receptor (EPCR), with a less prominent presence of TF.^47^ It remains to be established whether these differences are expressed at the protein level and influence the magnitude or nature of GBM-associated thrombosis, or whether they entail recruitment of host cells and translate into non-coagulant effects of the coagulation system such as invasion, inflammation, or angiogenesis.

The coagulome is also altered by oncogenic pathways in brain tumors in which systemic thrombosis is not a common occurrence. For example, in cells derived from neuronal malignancies (such as MB or ETMR), the expression of activated MET, SHH, or certain microRNA species regulate the expression of TF, PAR-1, and other coagulation-related factors^113^ (D’Asti and Rak, unpublished observations). In addition, the molecular subtypes that have recently redefined the classification and care in medulloblastoma (formerly a subset of PNET) are also associated with distinctive changes in the tumor coagulome, as measured by the levels of the respective transcripts in a large cohort of tumor samples. These tumors are highly vascular but are not known to provoke systemic thrombosis, and therefore changes in levels of TF and other coagulation effectors could have more subtle and context-related effects. For example, the coagulant phenotype of MB cells could contribute to the responses of these tumors to iatrogenic insults (e.g. surgery) and/or influence the tumor biology in other ways.^71^

## BIOLOGICAL AND THERAPEUTIC IMPLICATIONS OF CHANGES IN THE BRAIN TUMOR COAGULOME

The significance of studies on the role of the coagulation system in brain tumors is ultimately founded on their potential clinical utility. In this regard, coagulation-related events may contribute to outcomes through exacerbating thrombotic comorbidities,^29^ or as biomarkers of poor prognosis, impending relapse, or aggressive brain tumor biology.^104^,^108^ It is also possible that the pro-inflammatory and prometastatic effects of the TF pathway^34^,^114^,^115^ may have their reflection in metastasis of visceral malignancies to the brain or in infiltrative properties of primary brain tumors.^67^,^101^,^102^,^116^ In such cases, the addition of anticoagulant therapy could, at least in theory, mitigate these undesirable influences, offset iatrogenic coagulopathy, and possibly improve outcomes.

However, these approaches remain largely unexplored in spite of the increasing sophistication of anticoagulant and anti-platelet pharmacotherapy,^117^ and this may be due to several challenges. One important roadblock in this regard is the concern related to the perceived risk of intracranial bleeding that may accompany anticoagulation of patients with brain tumors.^118^ Such concerns are not borne out in the clinical experience with thromboprophylaxis in GBM,^29^ and can be reduced further by using agents with lower CNS bleeding risks. While this requires more extensive clinical analysis, it has been suggested that direct-acting oral anticoagulants (DOACs) may carry lower cranial bleeding risks than their conventional counterparts.^119^ There is also compelling preclinical evidence that certain anti-TF antibodies (e.g. 10H10) do not interfere with the hemostatic effects of the TF pathway, but rather selectively target the coagulation system signaling.^115^ In principle, such agents would be devoid of hemostatic side effects or bleeding risk and could have activity in settings where TF signaling plays a pathogenetic role. However, development of such agents or their analogues should proceed with caution, as several published anti-TF antibodies may retain some anticoagulant activity (e.g. 5G9) and would require thorough consideration of hemostatic safety. Nonetheless, the tempered enthusiasm for such explorations stems also from discouraging experiences with “generic” anticoagulation such as the use of vitamin K antagonists (VKAs) or low-molecular-weight heparins (LMWH), which produced inconsistent (or no) survival benefits in various cancer settings.^48^,^120^–^122^ The question is why?

Arguably, and due to the complexity of cancer coagulopathy, clinical explorations in this field have been conducted with an assumption of the fundamental similarity and hemostatic predominance of coagulation disorders in human cancers, and thus in the absence of molecular stratification, precision targeting, and biologically based personalization of the study design. Perhaps, one way to revisit these challenges and to formulate informative preclinical and clinical inquiries could be to bring forward two questions related to the aforementioned advances in cancer pathobiology: 1) Is there one or a spectrum of (molecular subtype-specific) coagulopathies in human brain tumors, and what are the implications of the latter possibility? 2) What are the disease subtype- and stage-related mechanisms, and what is the biological importance of coagulation system involvement in the progression of specific brain tumors? In other words, do specific coagulation effectors play a rate-limiting role throughout the disease, only at specific points in progression, or never in molecularly defined brain tumors? Is the role of the coagulation pathway the same or different at the time of tumor initiation, surgery, relapse, or progression?

These questions remain unresolved. One possibly informative example of the stage-specific role of the coagulation system in the progression of brain tumors could be derived from studies on the regulation of tumor initiation and on the exit from the state known as tumor dormancy. Both of these events rely upon the ability of cancer cells to assume TIC characteristics, which could be influenced by the coagulant microenvironment directly, or through the role of inflammation, angiogenesis, and tissue repair processes.^71^ In this regard, it is puzzling that full-blown GBMs are often diagnosed a surprisingly short time (4–10 months) after an apparently negative brain imaging.^123^ This is paradoxical as such a short period of genetic evolution time is difficult to reconcile with the genetic complexity of adult GBM,^124^ a feature which in other disease sites is known to take decades to develop.^59^ Therefore, it could be argued that the accumulation of genetic hits over the lifetime of an individual may lead to the formation of a population of dormant transformed cells in the brain without an apparent tumorigenesis;^101^ in a similar manner this is observed in the thyroid gland, prostate, or breast.^125^ If this is the case, it is of interest to know what might trigger the “awakening” of such pre-GBM dormant cells.^101^ In this regard, it is tempting to speculate that several case reports and small retrospective clinical studies suggesting a possible link between GBM and brain injury or scarring^126^ may in fact (implicitly) be pointing to coagulation system activation, which is a part of these processes. Could this mean that vascular events could bring about the “awakening” of dormant brain tumors?

Recent experimental studies seem to suggest that this is at least a theoretical possibility. Indeed, the experimental expression of TF in a dormant glioma cell line was found to provoke recruitment of inflammatory cells and intense neovascularization followed by tumor formation after prolonged latency time.^101^ Interestingly, cells isolated from such TF-expressing tumors harbored permanent changes in their genome and epigenome. Thus TF provoked formation of the inflammatory microenvironment, in which tumor cells evolved (epi) genetically resulting in their reduced reliance on TF for the ability to grow as aggressive lesions in secondary recipients.^101^ Similarly, experiments involving targeting of TF in advanced large lesions containing highly transformed cells was less effective than similar treatment of incipient tumors.^69^ These experiments are not definitive, but they do suggest that, as with many targeted agents, there may be a substantial but not infinite window of opportunity to target coagulation system effectors during progression of brain malignancies.

## SUMMARY

The coagulation system is a part of the regulatory network that integrates parenchymal cells with the vasculature and inflammatory responses. While blood clotting is the most studied manifestation of coagulation system activity, this is possibly a “tip” of the biological “iceberg” in the context of brain tumors, in which a unique coagulant milieu may play a pathogenetic role still to be fully characterized. We suggest that the repertoire of oncogenic drivers and the molecular diversity of primary and secondary brain tumors may result in a comparably diverse spectrum of coagulant perturbations with a unique potential for clinical consequences, worth exploring and possibly targeting. Indeed, it is the understanding of the possible diversity of brain tumor coagulopathies that represents an outstanding challenge.

## Abbreviations:

CNS: central nervous system
EGFR: epidermal growth factor receptor;
EGFRvIII: EGFR variant III;
ETMR: embryonal tumor with multilayered rosettes;
GBM: glioblastoma multiforme;
MB: medulloblastoma;
PAR: protease-activated receptor;
PBT: primary brain tumor;
PNET: primitive neuroectodermal tumor;
SBT: secondary brain tumor;
TF/F3: tissue factor/coagulation factor 3.
